# The use of synaptic biomarkers in cerebrospinal fluid to differentiate behavioral variant of frontotemporal dementia from primary psychiatric disorders and Alzheimer’s disease

**DOI:** 10.1186/s13195-024-01409-8

**Published:** 2024-02-14

**Authors:** Shreyasee Das, Marie-Paule E. van Engelen, Julie Goossens, Dirk Jacobs, Bram Bongers, Jay L. P. Fieldhouse, Yolande A. L. Pijnenburg, Charlotte E. Teunissen, Eugeen Vanmechelen, Inge M. W. Verberk

**Affiliations:** 1 Department of Laboratory Medicine, Neurochemistry Laboratory, Amsterdam, UMC location VrijeUniversiteit Amsterdam, Boelelaan 1117, Amsterdam, 1081 HV The Netherlands; 2 Neurology, Amsterdam UMC location VUmc, Alzheimer Center Amsterdam, VrijeUniversiteit Amsterdam, De Boelelaan 1118, Amsterdam, 1081 HZ The Netherlands; 3 Amsterdam Neuroscience, Neurodegeneration, De Boelelaan 1085, Amsterdam, 1081 HV The Netherlands; 4 ADx NeuroSciences, Technologiepark-Zwijnaarde 6, 9052 Gent, Belgium

**Keywords:** Synaptic biomarkers, Frontotemporal dementia, Primary psychiatric disorders, Differential diagnosis

## Abstract

**Background:**

Lack of early molecular biomarkers in sporadic behavioral variants of frontotemporal dementia (bvFTD) and its clinical overlap with primary psychiatric disorders (PPD) hampers its diagnostic distinction. Synaptic dysfunction is an early feature in bvFTD and identification of specific biomarkers might improve its diagnostic accuracy. Our goal was to understand the differential diagnostic potential of cerebrospinal fluid (CSF) synaptic biomarkers in bvFTD versus PPD and their specificity towards bvFTD compared with Alzheimer’s disease (AD) and controls. Additionally, we explored the association of CSF synaptic biomarkers with social cognition, cognitive performance, and disease severity in these clinical groups.

**Methods:**

Participants with probable bvFTD (*n* = 57), PPD (*n* = 71), AD (*n* = 60), and cognitively normal controls (*n* = 39) with available CSF, cognitive tests, and disease severity as frontotemporal lobar degeneration-modified clinical dementia rating scale (FTLD-CDR) were included. In a subset of bvFTD and PPD cases, Ekman 60 faces test scores for social cognition were available. CSF synaptosomal-associated protein 25 (SNAP25), neurogranin (Ng), neuronal pentraxin 2 (NPTX2), and glutamate receptor 4 (GluR4) were measured, along with neurofilament light (NfL), and compared between groups using analysis of covariance (ANCOVA) and logistic regression. Diagnostic accuracy was assessed using ROC analyses, and biomarker panels were selected using Wald’s backward selection. Correlations with cognitive measures were performed using Pearson’s partial correlation analysis.

**Results:**

NPTX2 concentrations were lower in the bvFTD group compared with PPD (*p* < 0.001) and controls (*p* = 0.003) but not compared with AD. Concentrations of SNAP25 (*p* < 0.001) and Ng (*p* < 0.001) were elevated in patients with AD versus those with bvFTD and controls. The modeled panel for differential diagnosis of bvFTD versus PPD consisted of NfL and NPTX2 (AUC = 0.96, CI: 0.93–0.99, *p* < 0.001). In bvFTD versus AD, the modeled panel consisted of NfL, SNAP25, Ng, and GluR4 (AUC = 0.86, CI: 0.79–0.92, *p* < 0.001). In bvFTD, lower NPTX2 (Pearson’s *r* = 0.29, *p* = 0.036) and GluR4 (Pearson’s *r* = 0.34, *p* = 0.014) concentrations were weakly associated with worse performance of total cognitive score. Lower GluR4 concentrations were also associated with worse MMSE scores (Pearson’s *r* = 0.41, *p* = 0.002) as well as with worse executive functioning (Pearson’s *r* = 0.36, *p* = 0.011) in bvFTD. There were no associations between synaptic markers and social cognition or disease severity in bvFTD.

**Conclusion:**

Our findings of involvement of NTPX2 in bvFTD but not PPD contribute towards better understanding of bvFTD disease pathology.

**Supplementary Information:**

The online version contains supplementary material available at 10.1186/s13195-024-01409-8.

## Background

Frontotemporal dementia (FTD) is a clinically, genetically, and pathologically heterogeneous disease and the second most common form of young-onset dementia after Alzheimer’s disease (AD) [1]. The most prevalent form is the behavioral variant of FTD (bvFTD), which is characterized by slowly progressive behavioral symptoms and impaired social cognition. In 30% of the bvFTD patients, the disease is caused by a pathogenic mutation (C9ORF72, MAPT, GRN), but the majority of cases are denoted sporadic bvFTD (70%) [2]. The lack of an early, disease-specific molecular biomarker in sporadic bvFTD together with its significant overlap in clinical symptoms with primary psychiatric disorders (PPD) and frontotemporal hypometabolic patterns on [^18^F]-2-deoxy-2-fluoro-D-glucose (FDG) positron emission tomography ([^18^F]FDG-PET) scans hampers diagnostic distinction. This leads to misdiagnosis in 50% of the cases with an average diagnostic delay of 6.4 years [3–5].

Evidence from post-mortem and clinical studies including pre-dementia stages show that loss of synaptic function is a predominant early feature in bvFTD and correlates with the level of cognitive impairment [6–14]. In accordance, a recent in vivo study using the [^11^C]UCB-J PET tracer to detect synaptopathy showed widespread frontotemporal loss of synapses in symptomatic bvFTD patients, which is related to disease severity [14]. Also, lower brain synaptic densities on [^18^F]UCB-H PET were found in the temporal brain regions involved in social cognition, highlighting the clinical relevance of synaptopathy in the disease pathophysiology of FTD [11].

In agreement, using cerebrospinal fluid (CSF) biomarkers as indicators for synapse health, differential synaptic concentrations were found in genetic forms of FTD [12]. Previous studies on synaptic involvement in FTD predominately assessed genetic FTD cases, had a small sample size, or did not include PPD as a control group, whereas the latter is the most challenging to distinguish from bvFTD in clinical practice [10–12]. Identification of specific CSF synaptic markers in sporadic bvFTD might improve diagnostic accuracy and aid in a better understanding of FTD pathophysiology. In addition, specific CSF-synaptic panels can provide endpoints in future clinical trials of sporadic bvFTD as they might correlate and reflect cognitive and social functioning.

In an attempt to explore the synaptic pathology in FTD and AD, we recently performed a pilot study where we found that concentrations of CSF synaptic biomarkers synaptosomal-associated protein 25 (SNAP25) and neurogranin (Ng) were elevated in FTD compared with controls, while those of neuronal pentraxin 2 (NPTX2) were lower than in controls, suggesting these could be valuable biomarkers for FTD [15]. SNAP25 is a pre-synaptic vesicle protein involved in neurotransmission, while Ng in the post-synapse regulates calcium ion influxes, and NPTX2 present extracellularly in the synaptic cleft maintains synaptic plasticity [15]. Simultaneously, another recent study suggested that patients with primary psychiatric disorders had significantly lower expression of the post-synaptic protein glutamate receptor 4 (GluR4) in CSF compared with cognitively normal controls and therefore could be useful as biomarkers for PDD [16]. GluR4 is primarily involved in excitatory signal transmission [15]. The axonal protein neurofilament light chain (NfL) has emerged as a promising fluid biomarker to distinguish bvFTD from PPD. Several studies have reported the potential of NfL as a biomarker that correlates with brain atrophy, neurodegeneration, and cognition in dementias [17–20] and also with other neuronal damage, e.g., due to stroke, amyotrophic lateral sclerosis, and multiple sclerosis [3, 21–24].

In this study, we aimed to assess the diagnostic performance of CSF synaptic biomarkers in sporadic bvFTD versus PPD and their added value compared with NfL as well as their specificity towards bvFTD compared with AD and controls. Secondly, we assessed the association of CSF synaptic biomarkers with social cognition, cognitive performance, and disease severity in bvFTD.

## Methods

### Participants

Patients with sporadic probable bvFTD, PPD, AD, and cognitively normal controls who visited the memory clinic of the Alzheimer Center Amsterdam between 2003 and 2021 were included in this study [25–27]. We included individuals aged 45–75 years with available CSF in the biobank and available clinical data. Individuals with AD were included in the case of AD-positive CSF biomarkers. BvFTD, PPD, and controls were excluded in the case of AD-positive CSF biomarkers. The study was approved by the Medical Ethical Committee of Amsterdam UMC. All participants provided informed consent and the study has been carried out by the Declaration of Helsinki.

### Diagnostic procedure

All participants had an extensive, standardized diagnostic assessment including clinical evaluation by a cognitive neurologist and/or old age psychiatrist, blood examination to exclude somatic causes, administered neuropsychological tests assessing five cognitive domains (attention, memory, speed, executive functioning, visuospatial functioning) [28, 29], lumbar puncture for CSF assessment of the AD biomarkers amyloid-beta42, total tau, and phosphorylated tau181 to determine positive or negative AD biomarkers status (cutoffs applied as published elsewhere), electroencephalography and neuroimaging- magnetic resonance imaging, and, if indicated, a [^18^F]FDG-PET scan [3, 30]. The diagnosis was concluded in a multidisciplinary meeting using consensus criteria for probable FTD, PPD (DSM-V), and AD [25, 31, 32]. Controls had no evidence of current or recent psychiatric disorders nor a neurodegenerative disorder. Psychiatric diagnoses included mood disorders (*n* =33), personality disorders (*n* = 4), autism spectrum disorder (*n* = 3), anxiety disorder (*n* = 4), functional disorder (*n* = 5), schizophrenia (*n* = 2), and other psychiatry (*n* = 10) [33]. The psychotropic medications the patients were taking included antidepressants, mood stabilizers (lithium), benzodiazepines, amphetamines (methylphenidate), antiepileptic and antipsychotic drugs, and cholinesterase inhibitors (rivastigmine).

### Measures for cognition, disease severity, mood and behavioral symptoms

Participants underwent standardized neuropsychological assessments covering the indicated cognitive domains (memory, attention, executive functioning, language, visuospatial functioning) [29]. For each cognitive domain, at least two tests were used to provide a reliable outcome on cognitive functioning (Table 1) [28]. Per test, a *z*-score was calculated, and, subsequently, all *z*-scores covering a specific cognitive domain were averaged into a cognitive domain *z*-score. Total cognitive score was calculated as an average *z*-score based on all five cognitive domains (memory, attention, executive functioning, language, visuospatial functioning, Table 1).

**Table 1:** The cognitive tests included to calculate the *z*-scores for each cognitive domain [29]

**Cognitive domains**	**Tests included**
Memory	Visual association test (VAT), Dutch version of the Rey auditory verbal learning test (RAVLT) with subtests of total immediate recall and delayed recall
Attention	Digit span forward, trail-making test (TMT) A, Stroop color-word test I and II
Executive functioning	Stroop color-word test III, digit span backwards, frontal assessment battery (FAB), letter fluency test (version D-A-T)
Language	Visual association test (VAT)—“naming,” category fluency animals
Visuospatial functioning	Visual object and space perception (VOSP) battery: number location, dot counting, fragmented letters
Total cognitive score	Memory, attention, executive functioning, language, visuospatial functioning

A subgroup of the bvFTD group (*n* = 13) and the PPD group (*n* = 9) completed a facial emotional recognition test (the Ekman 60 faces test). Disease severity in bvFTD was scaled according to the Frontotemporal Lobar Degeneration-Modified Clinical Dementia Rating (FTLD-CDR) Scale, using the sum-of-boxes score [34]. Also, participants completed the Geriatric Depression Scale (GDS) (mood), the Frontal Assessment Battery (FAB) (behavioral symptoms), and the Mini-Mental State Exam (MMSE) for global cognition. All tests and FTLD-CDR were completed within 12 months of the CSF withdrawal.

### CSF biomarker measurements

NfL was measured using a novel ELISA developed at ADx NeuroSciences, described elsewhere [35]. Synaptic protein Ng was measured using a commercial ELISA from EuroImmun, while the other synaptic proteins SNAP25, NPTX2, and GluR4 were measured using novel immunoassays developed and validated as per standardized protocols at ADx NeuroSciences, described in detail elsewhere [15, 36, 37]. The biomarkers are stable up to at least four freeze-thaw cycles in CSF. Thus, we measured SNAP25 and Ng in the first freeze-thaw cycle of the CSF samples, NfL in the second, NPTX2 in the third, and GluR4 in the final fourth freeze-thaw cycle. All clinical duplicate measurements were well within the range of 20% coefficient of variation (CV) for all immunoassays, except GluR4 which had slightly higher variability. The intermediate precision (average %CV) for each immunoassay of quality control samples was as follows: NfL—12%, SNAP25—4%, Ng—11%, NPTX2—15%, and GluR4—25%.

### Statistics

All statistical analyses were performed using IBM SPSS Statistics (v.28.0.1.1) or RStudio (v.4.0.3). The demographic differences between the diagnostic groups were assessed using a one-way analysis of variance (ANOVA) or chi-square test where appropriate. The CSF biomarker concentrations and cognitive test scores were log10 transformed to fit a normal distribution. Analysis of covariance (ANCOVA) models corrected for age, sex, and psychotropic medication use with post hoc pairwise comparisons was used to determine differences in biomarker concentrations between the clinical groups, with Bonferroni’s multiple comparison correction. Next, logistic regression analysis was performed to assess the association between the CSF biomarkers and diagnosis for the groups bvFTD versus PPD and bvFTD versus AD while controlling for the effect of age, sex, and psychotropic medication use. Additionally, we used Wald’s backward logistic regression on the biomarkers to select a biomarker panel for the group comparisons between bvFTD and AD or PPD. Receiver operating characteristics (ROC) curves were constructed for the CSF synaptic biomarkers and CSF NfL, as well as for the biomarker panels, not controlling for any potential confounders. For each ROC curve, the sensitivity and specificity were determined at Youden’s indices. Correlation between the CSF synaptic biomarkers and CSF NfL and cognitive test scores were assessed using Pearson’s partial correlation analysis controlling for age. Significance was defined as *p* < 0.05.

## Results

### Demographic characteristics

The demographic characteristics of the patients are detailed in Table 2. This cohort included 57 patients with bvFTD (age: 64 ± 8, female: 37%), 71 patients with PPD (age: 56 ± 9, female: 38%), 60 patients with AD (age: 66 ± 7, female: 45%), and 39 controls (age: 57 ± 8, female: 33%). The bvFTD and AD groups were significantly older than the PPD group and controls (*p* < 0.001 for all). No group differences were found for sex. Thirty percent of patients with bvFTD, 51% of those with PPD, 35% of the AD group, and 15% of the controls used psychotropic medication. Following expectations, psychotropic medication use was more frequent in the PPD group (*p* < 0.001) than in the control group.

**Table 2:** Demographic characteristics of the study cohort

**Diagnostic groups**	**bvFTD (*n* = 57)**	**PPD (*n* = 71)**	**AD (*n* = 60)**	**Controls (*n* = 39)**	***p*-value**
**Age**	64 (8)^b,d^	56 (9)^a,c^	66 (7) ^b,d^	57 (8) ^a,c^	< 0.001
**Female sex (%)**	21 (37%)	27 (38%)	27 (45%)	13 (33%)	0.737
**Psychotropic medication use (% Yes)**	16 (30%)	36 (51%)^d^	21 (35%)	5 (15%)^b^	0.014
**CSF biomarkers (pg/mL)**					
NfL	1630 (1052)^b,c,d^	369 (178)^a,c^	848 (332)^a,b,d^	337 (187)^a,c^	< 0.001
SNAP25	37 (32)^c^	33 (22)^c^	51 (19)^a,b,d^	28 (7)^c^	< 0.001
Ng	389 (312)^c^	351 (230)^c^	723 (1069)^a,b,d^	283 (107)^c^	< 0.001
NPTX2	401 (270)^b,d^	583 (300)^a^	477 (227)^b^	542 (240)^a^	< 0.001
GluR4	1100 (995)	1223 (1070)	1112 (510)	1070 (386)	0.456
**Cognitive domains**					
Memory	− 0.13 (0.6)^b,c,d^	0.41 (0.6)^a,c^	− 0.95 (0.7)^a,b,d^	0.73 (0.6)^a,c^	< 0.001
Attention	− 0.35 (1.0)^b,d^	0.14 (0.8)^a,c,d^	− 0.27 (0.8)^b,d^	0.53 (0.3)^a,b,c^	< 0.001
Executive functioning	− 0.44 (0.9)^b,d^	0.16 (0.6)^a,c,d^	− 0.43 (0.8)^b,d^	0.64 (0.4)^a,b,c^	< 0.001
Language	− 0.29 (0.7)^b,d^	0.32 (0.5)^a,c^	− 0.44 (0.9)^b,d^	0.60 (0.5)^a,c^	< 0.001
Visuospatial functioning	0.11 (0.5)^c^	0.19 (0.5)^c^	− 0.64 (1.1)^a,b,d^	0.34 (0.3)^c^	< 0.001
Total cognitive score	− 0.43 (0.8)^b,d^	0.2 (0.7)^a,c,d^	− 0.60 (0.7)^b,d^	0.60 (0.3)^a,b,c^	< 0.001
**Other tests**					
MMSE	24.3 (5)^b,d^	26.9 (2)^a,c^	20.7 (5)^b,d^	28.3 (1)^a,c^	< 0.001
Geriatric depression scale	3.2 (3)^b^	6.3 (4)^a,c,d^	2.9 (3)^b^	3.4 (3)^b^	< 0.001
Ekman 60 faces test	33.7 (7)^b^	42.7 (7)^a^	-	-	0.023
FTLD- CDR	7 (4)	-	-	-	-

Cognitive domain data are represented by *z*-scores. Other tests are represented as absolute scores. Missing data: GluR4: 3 bvFTD, 3 AD, 1 control; memory: 2 AD, 9 bvFTD, 3 PPD; attention: 2 bvFTD; executive functioning: 2 AD, 4 bvFTD, 2 PPD; visuospatial functioning: 22 AD, 25 bvFTD, 11 PPD, 4 controls; total cognitive score: 2 bvFTD; MMSE: 3 AD, 1 bvFTD, 1 PPD; geriatric depression scale: 7 AD, 11 bvFTD, 6 PPD, 2 controls; Ekman 60 faces test: 44 bvFTD, 62 PPD (not available for AD and controls); FTLD-CDR (available for bvFTD only): 2 bvFTD

*bvFTD*, behavioral frontotemporal dementia; *PPD*, primary psychiatric disorders; *AD*, Alzheimer’s disease; *NfL*, neurofilament light; *SNAP25*, synaptosomal-associated protein 25; *Ng*, neurogranin; *NPTX2*, neuronal pentraxin 2; *MMSE*, mini-mental state examination; *FTLD-CDR*, frontotemporal lobar degeneration-modified clinical dementia rating scale

Data represents the mean (SD) or *n* (%); ^a^*p* < 0.05 vs bvFTD, ^b^*p* < 0.05 vs PPD, ^c^*p* < 0.05 vs AD, ^d^*p* < 0.05 vs controls

Among the cognitive domain scores, the bvFTD group scored significantly (*p* < 0.05) lower than those with PPD for all domains except visuospatial functioning. Furthermore, patients with AD had significantly lower memory (*p* < 0.001) and visuospatial functioning (*p* = 0.004) scores than the bvFTD group. As expected, the bvFTD group had lower MMSE scores than PPD (*p* = 0.018) or controls (*p* = 0.004), while the AD group had the lowest MMSE scores, the difference being significant compared with PPD (*p* < 0.001) or controls (*p* < 0.001) but not bvFTD. Following expectations, the Ekman 60 faces test scores were lower in bvFTD patients compared with PPD patients (*p* = 0.012) (not available for AD and controls). All bvFTD patients in this cohort had mild to moderate FTD disease severity (FTLD-CDR ≤ 16).

### Differential concentrations of candidate CSF biomarkers across the diagnostic groups

**Fig. 1:**
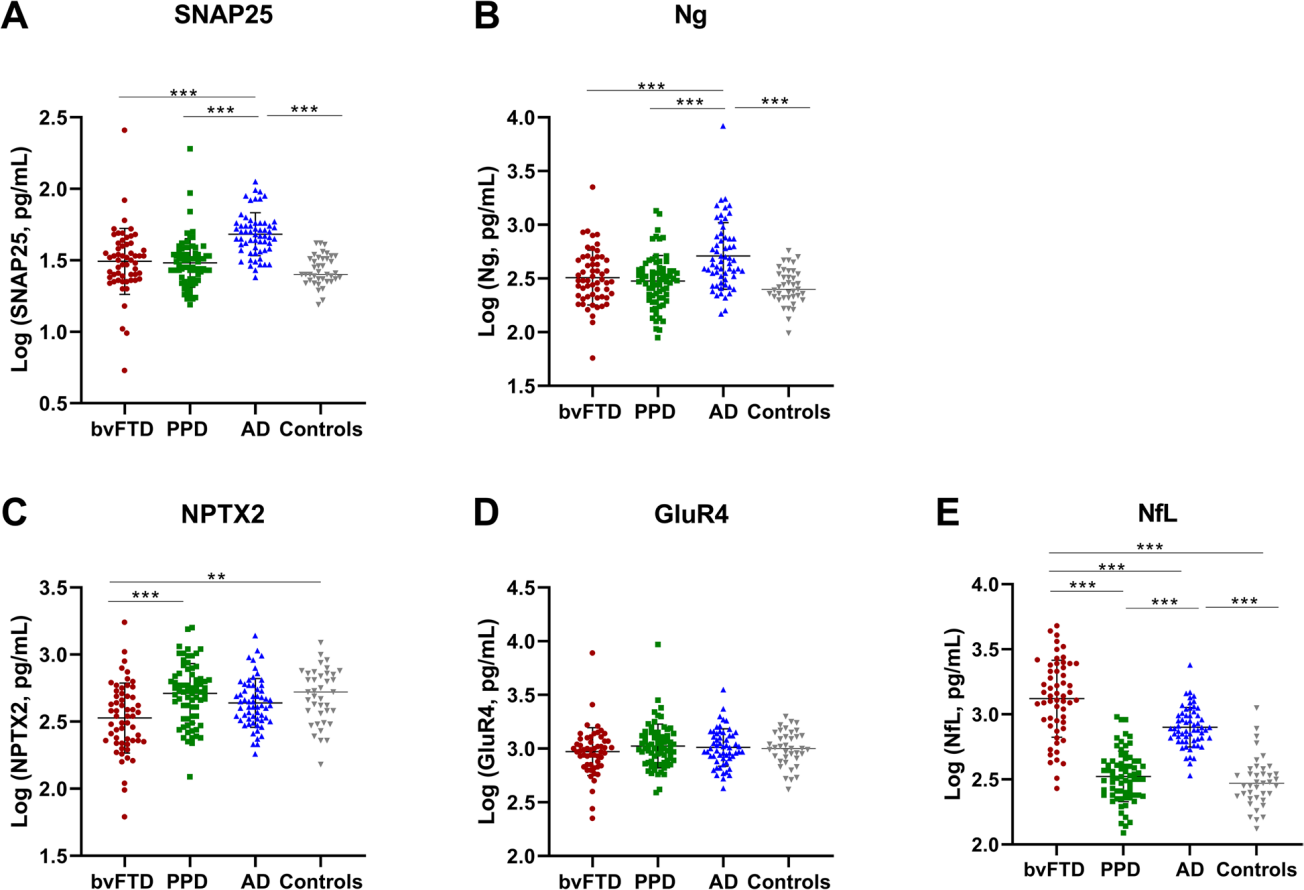
Concentrations of candidate CSF biomarkers across the diagnostic groups. Analysis of covariance (ANCOVA) models corrected for age, sex, and psychotropic medication use with post hoc pairwise comparisons were used to determine the log10 transformed biomarker differences between the clinical groups, with Bonferroni’s multiple comparison correction. **A** SNAP25. **B** Ng. **C** NPTX2. **D** GluR4. **E** NfL. SNAP25, synaptosomal*-*associated protein 25; Ng*,* neurogranin; NPTX2*,* neuronal pentraxin 2; GluR4, glutamate receptor 4; NfL, neurofilament light. bvFTD, behavioral frontotemporal dementia; PPD, primary psychiatric disorders; AD, Alzheimer’s disease. *p < 0.05, **p < 0.01, ***p < 0.001

The concentrations of the CSF biomarkers are presented in Table 2 and visualized in Fig. 1. Adjusted for age, sex, and psychotropic medication use, we found that NPTX2 concentrations were significantly lower in bvFTD compared with PPD (*p* < 0.001) and controls (*p* = 0.005). There was a trend towards lower average NPTX2 concentrations in bvFTD than those in the AD group, although this difference was not significant. SNAP25 and Ng concentrations were higher in AD compared with bvFTD, PPD, and controls (*p* < 0.001 for all). CSF GluR4 concentrations did not differ between the diagnostic groups. Furthermore, NfL concentrations were higher in bvFTD compared with AD, PPD, and controls (*p* < 0.001 for all). NfL did not differ between PPD and controls, while patients with AD had higher NfL concentrations compared with PPD and controls (*p* < 0.001 for both).

The logistic regression models for each biomarker controlled for age, sex, and psychotropic medication use, and their predictive value for bvFTD compared with PPD and AD are detailed in Table 3. Of the synaptic biomarkers, only NPTX2 had a significant predictive value for bvFTD versus PPD (odds ratio, OR: 0.997 [0.996, 0.999], *p* = 0.007). The synaptic biomarkers SNAP25 (OR: 0.966 [0.943, 0.990], *p* = 0.005) and Ng (OR: 0.998 [0.997, 0.999], *p* = 0.006) had significant diagnostic values for bvFTD versus AD.

**Table 3:** Predictive value of the CSF biomarkers for bvFTD versus PPD and AD

**Biomarker**	**bvFTD vs PPD**	**bvFTD vs AD**
	**OR (95% CI)**	**OR (95% CI)**
**NfL**	1.006 (1.003, 1.009)***	1.002 (1.001, 1.003)***
**SNAP25**	0.999 (0.986, 1.013)	0.966 (0.943, 0.990)**
**Ng**	1.000 (0.998, 1.001)	0.998 (0.997, 0.999)**
**NPTX2**	0.997 (0.996, 0.999)**	0.999 (0.997, 1.000)
**GluR4**	1.000 (0.999, 1.000)	1.000 (0.999, 1.000)

The logistic regression models for each biomarker are controlled for confounders’ age, sex, and psychotropic medication use. *OR*, odds ratio; *NfL*, neurofilament light; *SNAP25*, synaptosomal-associated protein 25; *Ng*, neurogranin; *NPTX2*, neuronal pentraxin 2; *GluR4*, glutamate receptor 4; *bvFTD*, behavioral frontotemporal dementia; *PPD*, primary psychiatric disorders; *AD*, Alzheimer’s disease. *p < 0.05, **p < 0.01, ***p < 0.001

### Diagnostic performance of the candidate CSF biomarkers

**Fig. 2:**
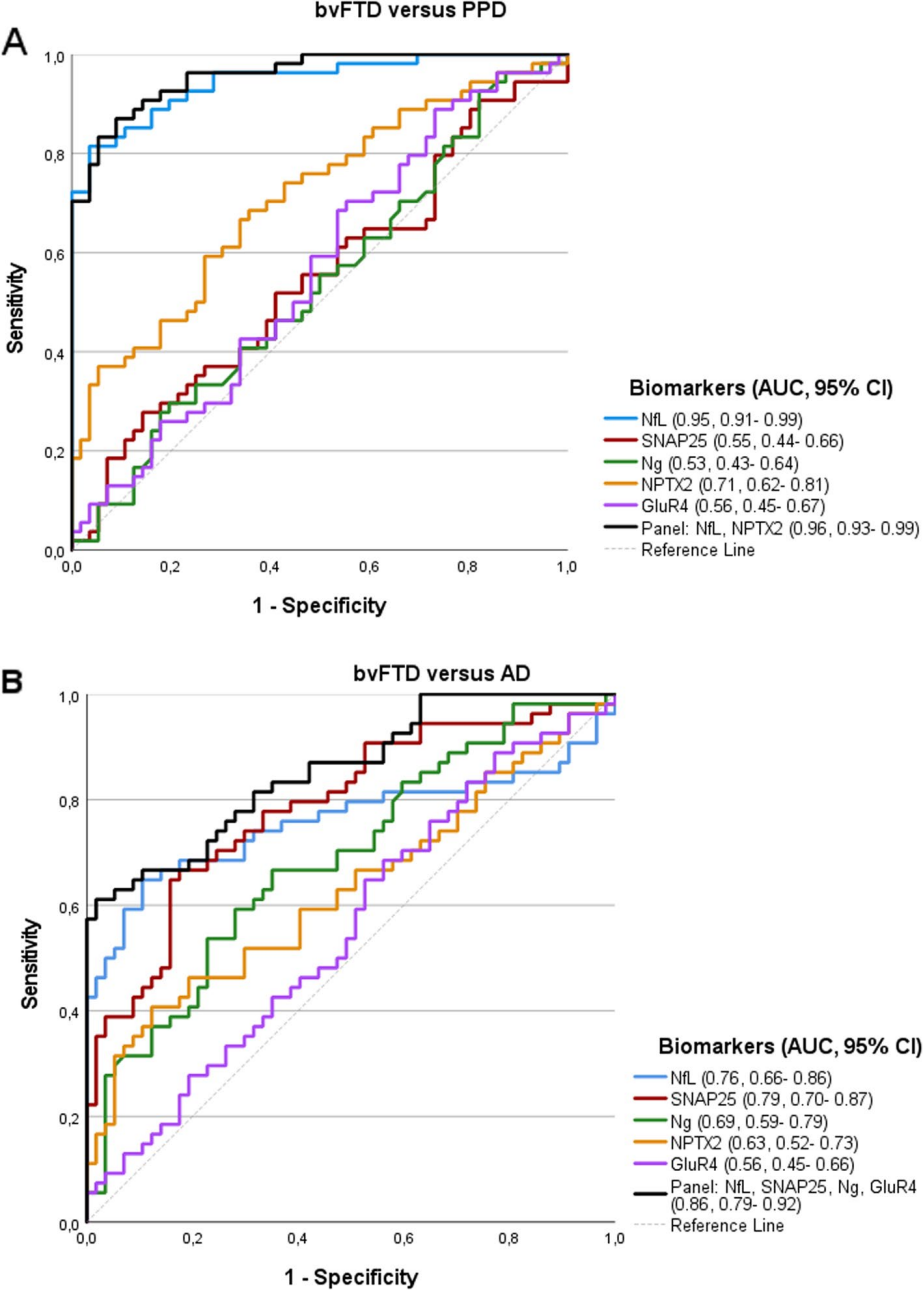
ROC curves showing the differentiation accuracy between the diagnostic groups of biomarkers in CSF. **A** bvFTD versus PPD, Panel-NfL, NPTX2. **B** bvFTD versus AD, Panel-NfL, SNAP25, Ng, GluR4. AUCs are shown for biomarkers alone. bvFTD, behavioral variant frontotemporal dementia, PPD, primary psychiatric disorders; AD, Alzheimer’s disease; AUC, area under curve; ROC, receiver operating characteristics; NfL, neurofilament; SNAP25, synaptosomal*-*associated protein 25; Ng, neurogranin; NPTX2, neuronal pentraxin 2; GluR4, glutamate receptor 4

We further investigated the diagnostic potential of the CSF biomarkers, stand-alone, as well as statistically selected panels, uncorrected for possible confounders (Table 4, Fig. 2).

**Table 4:** The AUC of the measured biomarkers as determined using ROC analysis

**Diagnostic potential of the CSF biomarkers and selected diagnostic panel**								
	**bvFTD vs PPD**				**bvFTD vs AD**			
**Biomarker**	**AUC**	**CI**	**Specificity (%)**	**Sensitivity (%)**	**AUC**	**CI**	**Specificity (%)**	**Sensitivity (%)**
**NfL**	0.95***	0.91–0.99	81.0	95.8	0.76***	0.66–.86	88.3	63.1
**SNAP25**	0.55	0.44–0.66	49.1	64.8	0.79***	0.70–0.87	81.7	68.4
**Ng**	0.53	0.43–0.64	53.0	52.1	0.69**	0.59–0.79	65.0	66.7
**NPTX2**	0.71***	0.62–0.81	70.2	66.2	0.63*	0.52–0.73	88.3	42.1
**GluR4**	0.56	0.45–0.67	70.4	46.5	0.56	0.45–0.66	44.0	68.5
**Panel**	0.96***	0.93-0.99	87.7	91.5	0.86***	0.79–0.92	98.2	61.1

Wald’s backward logistic regression was used to select the panel of CSF biomarkers. The biomarker panels selected were as follows: bvFTD vs PPD–NfL, NPTX2; bvFTD vs AD- NfL, SNAP25, Ng, GluR4. AUCs are shown for biomarkers alone. *bvFTD*, behavioral variant frontotemporal dementia; *PPD*, primary psychiatric disorders; *AD*, Alzheimer’s disease; *AUC*, area under curve; *ROC*, receiver operating characteristics; *CI*, confidence interval; *NfL*, neurofilament; *SNAP25*, synaptosomal-associated protein 25; *Ng*, neurogranin; *NPTX2*, neuronal pentraxin 2. *p < 0.05, **p < 0.01, ***p < 0.001

#### bvFTD versus PPD

Among the synaptic biomarkers, NPTX2 had the highest AUC (AUC= 0.72, CI: 0.63–0.81, *p* < 0.001) to discriminate bvFTD from PPD. SNAP25, Ng, and GluR4 were not predictive of the diagnostic group. The AUC of NfL (AUC= 0.95, CI: 0.91–0.99, *p* < 0.001) was higher than that of the synaptic proteins. The biomarker panel to differentiate bvFTD from PPD, selected using Wald’s backward selection among the candidate biomarkers consisted of NfL and NPTX2 (AUC =0.96, CI: 0.93–0.99, *p* < 0.001).

#### bvFTD versus AD

Among the synaptic biomarkers, SNAP25 had the highest AUC (AUC = 0.79, CI: 0.70–0.87, *p* < 0.001), followed by Ng (AUC = 0.69, CI: 0.59–0.79, *p* = 0.001) and NPTX2 (AUC = 0.63, CI: 0.52–0.73, *p* = 0.019) to discriminate bvFTD from AD. GluR4 was not predictive between these two diagnostic groups. The AUC of NfL (AUC= 0.75, CI: 0.65–0.84, *p* < 0.001) was higher than all synaptic biomarkers except SNAP25. The selected biomarker panel using Wald’s backward selection method, for differential diagnosis of bvFTD from AD, consisted of NfL, SNAP25, Ng, and GluR4 (AUC = 0.86, CI: 0.79–0.92, *p* < 0.001).

### Associations of the CSF synaptic biomarkers and NfL with cognition and disease severity

**Fig. 3:**
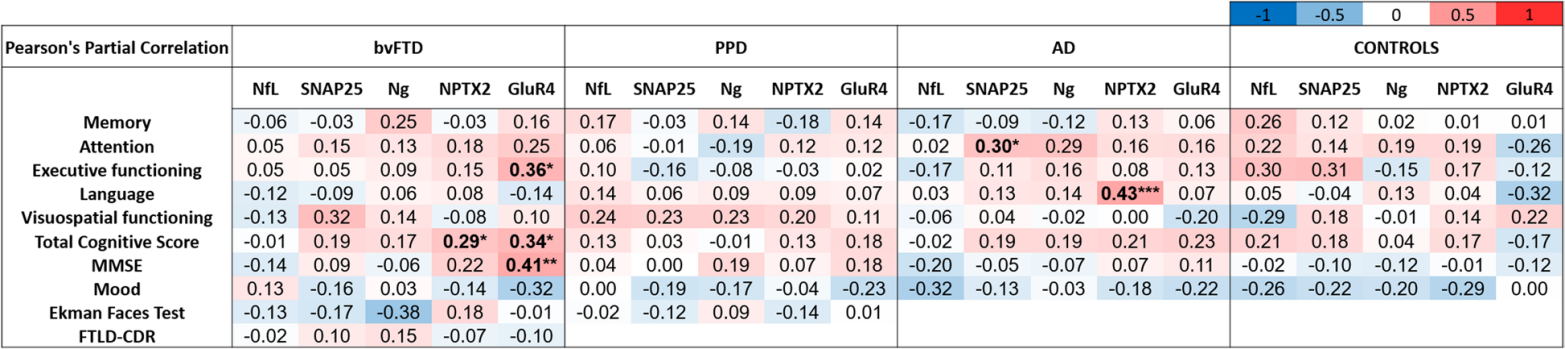
Correlation matrix of the fluid biomarkers to cognitive test performance and social test scores in patients with bvFTD, PPD, AD, and controls. The associations are shown as Pearson’s partial correlations, controlling for age. bvFTD, behavioral variant frontotemporal dementia; PPD, primary psychiatric disorders; AD, Alzheimer’s disease; NfL, neurofilament light; SNAP25, synaptosomal-associated protein 25; Ng, neurogranin; NPTX2, neuronal pentraxin 2; GluR4, glutamate receptor 4; MMSE, mini-mental state examination; FTLD-CDR, frontotemporal lobe dementia-cognitive dementia rating. *p < 0.05, **p < 0.01, ***p < 0.001

The correlations of the CSF biomarkers with cognition and disease severity are shown in Fig. 3, Supplementary Figure 1, and Supplementary Figure 2. There were no associations between any of the synaptic markers and social cognition (Ekman 60 faces test) or disease severity in bvFTD. Lower CSF concentrations of NPTX2 (Pearson’s *r* = 0.29, *p* = 0.036) and GluR4 (Pearson’s *r* = 0.34, *p* = 0.014) were weakly associated with worse performance of total cognitive score in bvFTD. Furthermore, lower GluR4 concentrations were also moderately associated with worse absolute MMSE scores in bvFTD (Pearson’s *r* = 0.41, *p* = 0.002) and weakly associated with worse executive functioning (Pearson’s *r* = 0.36, *p* = 0.011). In patients with AD, lower NPTX2 concentrations were moderately associated with worse performance scores on the cognitive domain language (Pearson’s *r* = 0.43, *p* < 0.001). Counterintuitively, in AD, higher CSF SNAP25 associated weakly with better performance on the domain attention (Pearson’s *r* = 0.30, *p* = 0.022). No significant correlations were detected between the CSF biomarkers and cognitive scores in patients with PPD or in controls. In the bvFTD and PPD groups, there were no significant correlations between cognitive scores and the generated biomarker panels (bvFTD_PPD, bvFTD_AD, Supplementary Figure 1). In the AD group, the panel bvFTD_AD correlated weakly with attention (Pearson’s *r* = − 0.30, *p* = 0.026), and in controls, the panel bvFTD_PPD correlated moderately with visuospatial functioning (Pearson’s *r* = − 0.42, *p* = 0.012).

## Discussion

In this study, we found differential concentrations of CSF synaptic markers between bvFTD, PPD, AD, and controls in which reduced NPTX2 concentrations were bvFTD specific, and increased concentrations of SNAP25 and Ng were AD specific. Adding NPTX2 to NfL in the biomarker panel to distinguish bvFTD and PPD patients provided added diagnostic value, although limited. Synaptic biomarker concentrations did not correlate with social cognition nor disease severity in FTD and showed weak correlations with cognitive performance. These results indicate that NPTX2, alongside NfL, may provide further insights into bvFTD pathophysiology, although it is relatively less suitable clinically as a diagnostic biomarker.

NfL is a reliable biomarker of neuroaxonal damage and is used for the diagnosis (although limited due to its non-disease specificity), prognosis, and monitoring of treatment response in several neurodegenerative conditions [38]. Several clinical reports have asserted the association of NfL with grey matter and hippocampal atrophy, neuronal impairment, and loss of cognition in neurodegenerative dementias [17–20]. Accumulating clinical evidence further suggests that NfL is a promising biomarker in clinical settings to differentiate patients with bvFTD from PPD [39]. However, high levels of NfL are not specific to bvFTD as this biomarker can be strongly elevated in several other neurodegenerative conditions as well [2, 40, 41]. Furthermore, clinical reports suggest that synaptic dysfunction precedes atrophy in patients with FTD [11, 14], which highlights the need for novel biomarkers that may aid in earlier and more accurate diagnosis of bvFTD over PPD.

Our findings of lower NPTX2 concentrations in sporadic FTD compared with PPD and controls are in line with our previous pilot study and with a recent study among patients with genetic forms of FTD, in which symptomatic mutation carriers showed lower concentrations of CSF NPTX2 compared with controls [13]. NPTX2 has also recently been identified as a promising biomarker for progression in genetic FTD [13]. NPTX2 is involved in the formation and stabilization of synapses, facilitating proper communication between neurons in the brain, and plays a crucial role in synaptic function and plasticity [42]. Studies have shown decreased synaptic density in the brains of FTD patients, particularly in brain regions affected by the disease including the salient network, inducing the characteristic impaired social cognition that is a hallmark feature of FTD [11]. Experimental evidence further suggests that downregulation of NPTX2 may lead to increased complement-mediated microglial activation, thereby causing abnormal elimination of synapses [43]. The downregulation of NPTX2 in both genetic and sporadic bvFTD may thus reflect a shared pathophysiology within the FTD disease heterogeneity and suggest that NPTX2 may play a crucial role in the pathogenesis of FTD by contributing to synaptic dysfunction [42]. Further investigation of NPTX2 and its mechanisms in FTD could provide valuable insights into the disease mechanisms and potentially lead to the development of novel therapeutic strategies targeting synaptic dysfunction.

While it has been reported elsewhere that CSF concentration of GluR4 is decreased in patients with mood disorder and schizophrenia compared with healthy controls [16], we did not find any diagnostic significance of this biomarker in this cohort. This might be due to our heterogeneous sample of PPD, including various subtypes such as mood disorders, personality disorders, autism spectrum disorder, anxiety disorder, functional disorder, schizophrenia, and other psychiatry, of which individual group levels did not reach statistically significant thresholds of lower GluR4.

Synaptic pathology is a shared mechanism across diseases, yet evidence presented here and elsewhere indicates that synaptic proteins participate differentially in various disease pathogeneses, underscoring the distinct impairments in synaptic functionality across diseases. For example, the AD-specific increase of CSF SNAP25 and Ng compared with bvFTD that we reported, corroborates previous findings [44, 45]. Ng is a postsynaptic protein that is important for maintaining synaptic plasticity and regulating calcium ion influxes, while SNAP25 is a presynaptic protein that plays a crucial role in synaptic vesicle fusion and neurotransmitter release, and both these proteins have been shown to play a key role in AD disease pathophysiology, although they may be less clinically relevant for FTD [10, 45–48]. While brain regions affected in AD, i.e., the hippocampus and cortex have a high expression of Ng, the anatomical distribution of SNAP25 is not well known, although it is expressed in the cortex [49–52]. Thus, a possible hypothesis for the increased concentrations of synaptic proteins SNAP25 and Ng in AD but not in bvFTD could be due to the topography of brain atrophy they reflect [45].

In our cohort, bvFTD patients performed worse on social cognition testing compared with PPD, but no association was found with CSF synaptic markers which might be due to the limited test scores available per diagnostic group, i.e., only 13 for bvFTD and 9 for PPD. A previous study, using [^18^F]UCBH-PET as a tracer for synaptic vesicle protein 2A (SV2A) which reflects synaptic density, showed a trend for synaptic loss in the temporal social brain in bvFTD, highlighting the clinical relevance of synaptopathy in disease pathophysiology of FTD [14]. Further studies assessing CSF synaptic markers might elucidate if SV2A is a superior synaptic marker in CSF correlating with social cognition in larger patient groups.

The association of the synaptic biomarkers with other cognitive functioning was absent or only moderate to weak in bvFTD and AD, while there were no correlations found in patients with PPD or with controls. Moreover, we did not detect any association of the synaptic proteins or NfL with FTLD-CDR disease severity scores [53, 54]. One plausible reason could be that the cohort included a homogenous sample of bvFTD patients with mild to moderate bvFTD disease severity (FTLD-CDR ≤ 16). The direction of correlation detected in the AD group between SNAP25 and attention was counterintuitive, and thus follow-up studies with greater statistical power are necessary to evaluate these findings.

The strengths of this study lie in including the assessment of concentrations of CSF synaptic proteins involved in several synaptic functions both upstream and downstream of the synapse, providing insight into pathophysiological mechanisms. Additionally, we included a well-phenotyped diverse PPD sample as a comparative group, which is the most important and clinically challenging to differentially diagnose from bvFTD, resembling clinical practice. There are also some limitations. For example, the odds ratios of the synaptic biomarkers for diagnostic distinction of bvFTD versus PPD and AD were modest and the clinical relevance of these biomarkers, particularly NPTX2, demands to be evaluated in larger cohorts. Since the PPD sample was heterogeneous, disease-specific synaptic concentrations in PPD could not be assessed and should be included in future studies. Furthermore, the sample sizes for cognitive test scores were limited, such as for social cognition (Ekman 60 faces test), disease severity (FTLD-CDR), and the domain visuospatial functioning.

## Conclusions

We conclude that synaptic biomarker NPTX2 has additional, although limited diagnostic value to NfL in the differential diagnosis of bvFTD versus PPD. Our findings contribute insight into disease-specific mechanisms in bvFTD, by showing the bvFTD-specific decrease in concentrations of NPTX2. Furthermore, given that NfL likely reflects neuronal atrophy [17, 18], it is a clinically relevant biomarker at a progressed stage of the disease. Further investigation of NPTX2 and its mechanisms in bvFTD could provide valuable insights into the disease mechanisms for early diagnosis and prognosis, as well as potentially lead to the development of novel therapeutic strategies.

### Supplementary Information

**Additional file 1: Supplementary Figure 1.** Correlation matrix of the fluid biomarkers to cognitive test performance and social test scores in patients with bvFTD, PPD, AD, and controls. The associations are shown as Pearson’s partial correlations, controlling for age. bvFTD: behavioral variant frontotemporal dementia, PPD: primary psychiatric disorders, AD: Alzheimer’s disease, NfL: neurofilament light, SNAP25: synaptosomal associated protein 25, Ng: neurogranin, NPTX2: neuronal pentraxin 2, GluR4: Glutamate receptor 4, MMSE: mini-mental state examination, FTLD-CDR: frontotemporal lobe dementia- cognitive dementia rating. Panel bvFTD_PPD: NfL, NPTX2, Panel bvFTD_AD: NfL, SNAP25, Ng, GluR4 (both differential diagnostic panels selected using backward logistic regression models).*p <0.05, **p <0.01, ***p <0.001

**Additional file 2: Supplementary Figure 2.** Visualization of correlations between biomarkers and cognitive test scores. A) bvFTD: NPTX2 versus total cognitive score, B) bvFTD: GluR4 versus total cognitive score, C) bvFTD: GluR4 versus executive functioning, D) bvFTD: GluR4 versus MMSE scores, E) AD: NPTX2 versus language and F) AD: SNAP25 versus attention. bvFTD: behavioral variant frontotemporal dementia, AD: Alzheimer’s diseaseSNAP25: synaptosomal associated protein 25, NPTX2: neuronal pentraxin 2, GluR4: Glutamate receptor 4, MMSE: mini-mental state examination.

## Data Availability

Anonymized data can be made available upon reasonable request and consultation with the involved authorities. Additional datasets generated during and/or analyzed during the current study are available from the corresponding author upon reasonable request.

## Abbreviations

AD: Alzheimer’s disease
ANCOVA: Analysis of covariance
ANOVA: Analysis of variance
AUC: Area under curve
bvFTD: Behavioral variant frontotemporal dementia
CSF: Cerebrospinal fluid
CV: Coefficient of variation
FAB: Frontal assessment battery
FTD: Frontotemporal dementia
FTLD-CDR: Frontotemporal lobar degeneration-modified clinical dementia rating
GDS: Geriatric depression scale
GluR4: Glutamate receptor 4
MMSE: Mini-mental state examination
NfL: Neurofilament light
Ng: Neurogranin
NPTX2: Neuronal pentraxin 2
OR: Odds ratio
PPD: Primary psychiatric disorders
RAVLT: Rey auditory verbal learning test
ROC: Receiver operating characteristics
SNAP25: Synaptosomal-associated protein of 25 kDa
TMT: Trail making test
VAT: Visual association test
VOSP: Visual object and space perception
