# Supplementary material for: The use of synaptic biomarkers in cerebrospinal fluid to differentiate behavioral variant of frontotemporal dementia from primary psychiatric disorders and Alzheimer’s disease
Source: Alzheimers Res Ther. 2024 Feb 14;16:34. doi: 10.1186/s13195-024-01409-8 (PMC10865562; doi:10.1186/s13195-024-01409-8)
Supplement: Supplementary file 1 — Additional file 1: Supplementary Figure 1. Correlation matrix of the fluid biomarkers to cognitive test performance and social test scores in patients with bvFTD, PPD, AD, and controls. The associations are shown as Pearson’s partial correlations, controlling for age. bvFTD: behavioral variant frontotemporal dementia, PPD: primary psychiatric disorders, AD: Alzheimer’s disease, NfL: neurofilament light, SNAP25: synaptosomal associated protein 25, Ng: neurogranin, NPTX2: neuronal pentraxin 2, GluR4: Glutamate receptor 4, MMSE: mini-mental state examination, FTLD-CDR: frontotemporal lobe dementia- cognitive dementia rating. Panel bvFTD_PPD: NfL, NPTX2, Panel bvFTD_AD: NfL, SNAP25, Ng, GluR4 (both differential diagnostic panels selected using backward logistic regression models).*p <0.05, **p <0.01, ***p <0.001 [file 13195_2024_1409_MOESM1_ESM.docx]

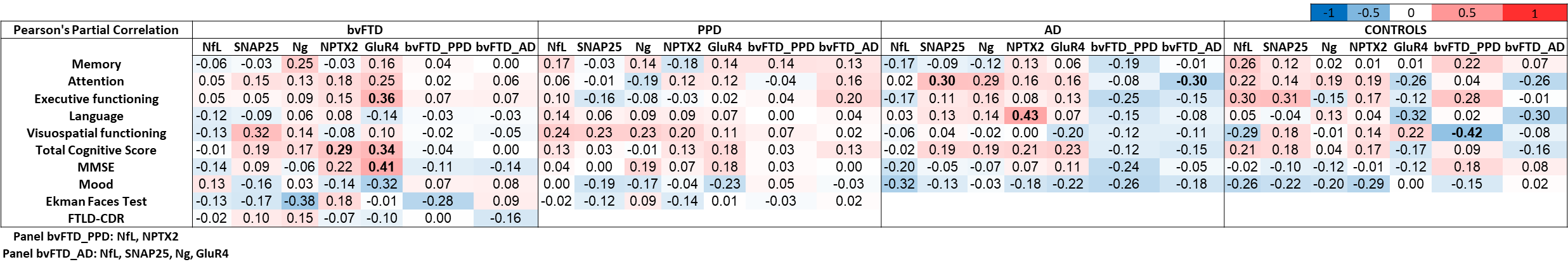


Supplementary Figure 1: Correlation matrix of the fluid biomarkers to cognitive test performance and social test scores in patients with bvFTD, PPD, AD, and controls. The associations are shown as Pearson’s partial correlations, controlling for age. bvFTD: behavioral variant frontotemporal dementia, PPD: primary psychiatric disorders, AD: Alzheimer’s disease, NfL: neurofilament light, SNAP25: synaptosomal associated protein 25, Ng: neurogranin, NPTX2: neuronal pentraxin 2, GluR4: Glutamate receptor 4, MMSE: mini-mental state examination, FTLD-CDR: frontotemporal lobe dementia- cognitive dementia rating. Panel bvFTD_PPD: NfL, NPTX2, Panel bvFTD_AD: NfL, SNAP25, Ng, GluR4 (both differential diagnostic panels selected using backward logistic regression models).*p <0.05, **p <0.01, ***p <0.001


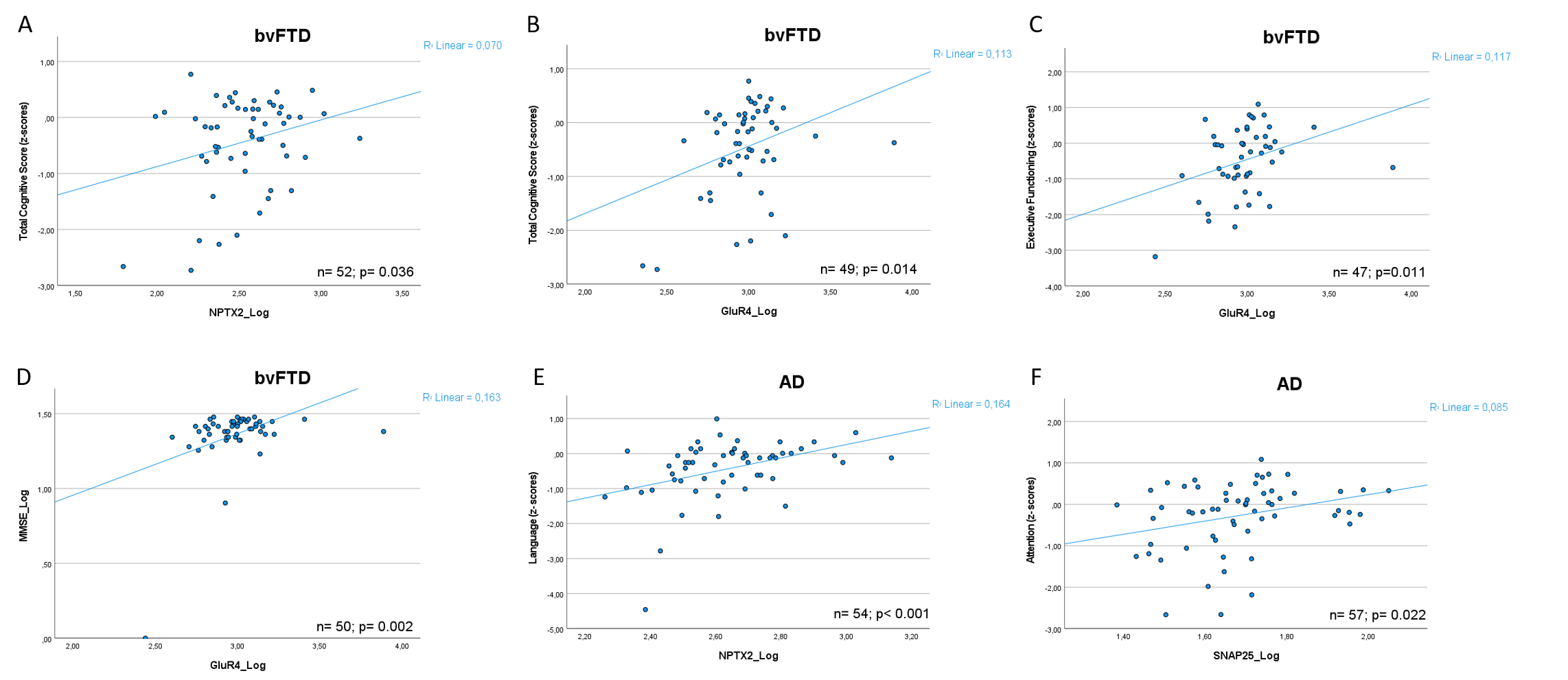


Supplementary Figure 2: Visualization of correlations between biomarkers and cognitive test scores. A) bvFTD: NPTX2 versus total cognitive score, B) bvFTD: GluR4 versus total cognitive score, C) bvFTD: GluR4 versus executive functioning, D) bvFTD: GluR4 versus MMSE scores, E) AD: NPTX2 versus language and F) AD: SNAP25 versus attention. bvFTD: behavioral variant frontotemporal dementia, AD: Alzheimer’s diseaseSNAP25: synaptosomal associated protein 25, NPTX2: neuronal pentraxin 2, GluR4: Glutamate receptor 4, MMSE: mini-mental state examination.
