# Supplementary figures and images for: The use of synaptic biomarkers in cerebrospinal fluid to differentiate behavioral variant of frontotemporal dementia from primary psychiatric disorders and Alzheimer’s disease
Source: Alzheimers Res Ther. 2024 Feb 14;16:34. doi: 10.1186/s13195-024-01409-8 (PMC10865562; doi:10.1186/s13195-024-01409-8)

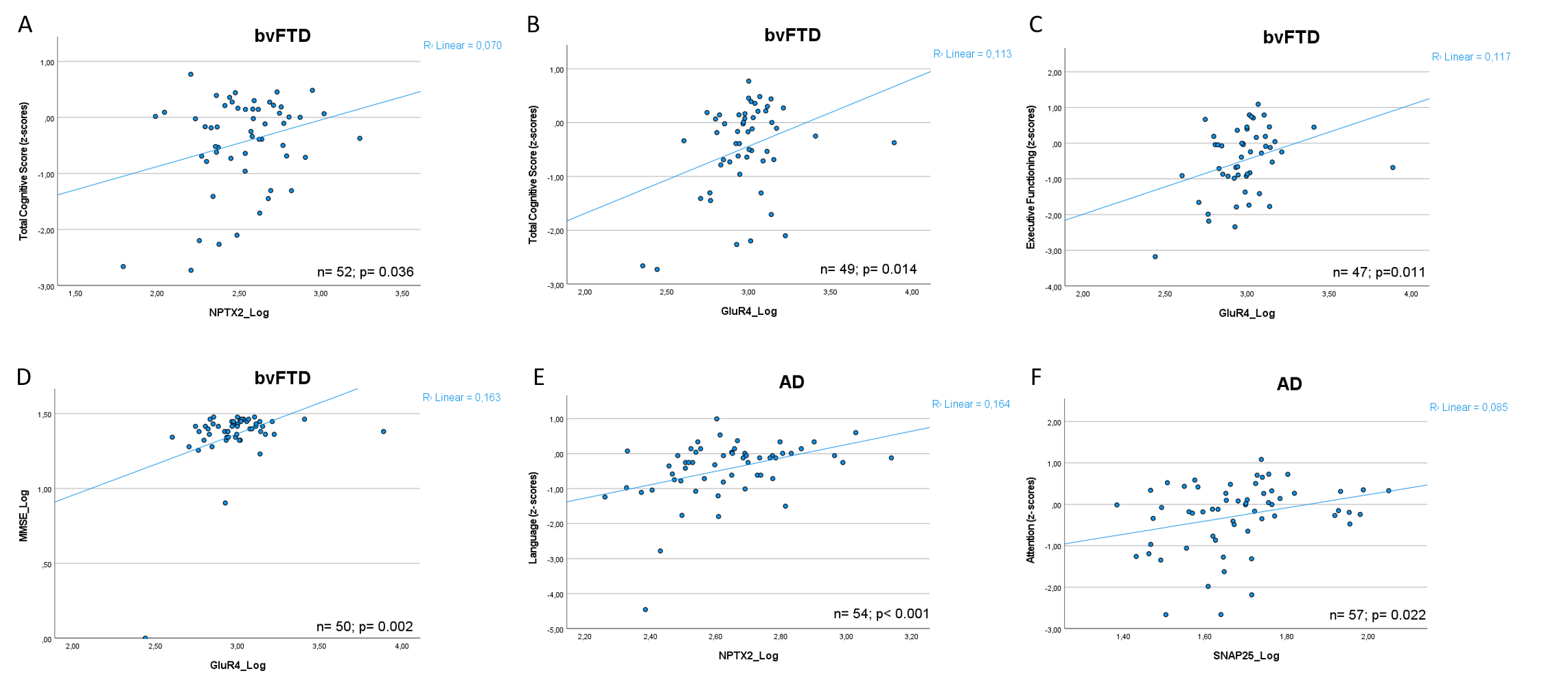

Supplement: Supplementary file 2 — Additional file 2: Supplementary Figure 2. Visualization of correlations between biomarkers and cognitive test scores. A) bvFTD: NPTX2 versus total cognitive score, B) bvFTD: GluR4 versus total cognitive score, C) bvFTD: GluR4 versus executive functioning, D) bvFTD: GluR4 versus MMSE scores, E) AD: NPTX2 versus language and F) AD: SNAP25 versus attention. bvFTD: behavioral variant frontotemporal dementia, AD: Alzheimer’s diseaseSNAP25: synaptosomal associated protein 25, NPTX2: neuronal pentraxin 2, GluR4: Glutamate receptor 4, MMSE: mini-mental state examination. [file 13195_2024_1409_MOESM2_ESM.png]
